# Qualitative analysis of reasons for hospitalization for severe hypoglycemia among older adults with diabetes

**DOI:** 10.1186/s12877-021-02268-w

**Published:** 2021-05-17

**Authors:** Weronika E. Pasciak, David N. Berg, Emily Cherlin, Terri Fried, Kasia J. Lipska

**Affiliations:** 1 Netter School of Medicine at Quinnipiac University, New Haven, CT USA; 2 Department of Psychiatry, Yale School of Medicine, New Haven, CT USA; 3 Yale School of Public Health and Yale Global Health Leadership Initiative, New Haven, CT USA; 4 Clinical Epidemiology Research Center, VA Connecticut Healthcare System, West Haven, CT USA; 5 Department of Internal Medicine, Division of Geriatrics, Yale School of Medicine, New Haven, CT USA; 6 Department of Internal Medicine, Section of Endocrinology, Yale School of Medicine, New Haven, CT USA

**Keywords:** Hospitalization for hypoglycemia, Older adults with diabetes, Qualitative study

## Abstract

**Background:**

Hospital admissions for severe hypoglycemia are associated with significant healthcare costs, decreased quality of life, and increased morbidity and mortality, especially for older adults with diabetes. Understanding the reasons for hypoglycemia hospitalization is essential for the development of effective interventions; yet, the causes and precipitants of hypoglycemia are not well understood.

**Methods:**

We conducted a qualitative study of non-nursing home patients aged 65 years or older without cognitive dysfunction admitted to a single tertiary-referral hospital with diabetes-related hypoglycemia. During the hospitalization, we conducted one-on-one, in-depth, semi-structured interviews to explore: (1) experiences with diabetes management among patients hospitalized for severe hypoglycemia; and (2) factors contributing and leading to the hypoglycemic event. Major themes and sub-themes were extracted using the constant comparative method by 3 study authors.

**Results:**

Among the 17 participants interviewed, the mean age was 78.9 years of age, 76.5% were female, 64.7% African American, 64.7% on insulin, and patients had an average of 13 chronic conditions. Patients reported: (1) surprise at hypoglycemia despite living with diabetes for many years; (2) adequate support, knowledge, and preparedness for hypoglycemia; (3) challenges balancing a diet that minimizes hyperglycemia and prevents hypoglycemia; (4) the belief that hyperglycemia necessitates medical intervention, but hypoglycemia does not; and (5) tension between clinician-prescribed treatment plans and self-management based on patients’ experience. Notably, participants did not report the previously cited reasons for hypoglycemia, such as food insecurity, lack of support or knowledge, or treatment errors.

**Conclusions:**

Our findings suggest that some hypoglycemic events may not be preventable, but in order to reduce the risk of hypoglycemia in older individuals at risk: (1) healthcare systems need to shift from their general emphasis on the avoidance of hyperglycemia towards the prevention of hypoglycemia; and (2) clinicians and patients need to work together to design treatment regimens that fit within patient capacity and are flexible enough to accommodate life’s demands.

**Supplementary Information:**

The online version contains supplementary material available at 10.1186/s12877-021-02268-w.

## Background

Historically, professional organizations have emphasized the central role of glycemic control (e.g., hemoglobin A1c (HbA1c) < 7%) in diabetes management in order to reduce the risk of long-term complications of the disease [1]. However, a growing recognition of the burden of severe hypoglycemia has begun to shift diabetes care toward a greater emphasis on prevention of adverse drug events (ADEs) associated with diabetes treatment [1]. Emergency department (ED) visits for severe hypoglycemia are now more common than those for hyperglycemic crisis [2] and are associated with significant healthcare costs [3]. Alarmingly, the reported rates of severe hypoglycemia have not fallen for the past 20 years [4] and current surveillance continues to underestimate its overall burden [5]. Severe hypoglycemia, defined as an event requiring the assistance of another person [6], is associated with significant morbidity and greater risk of death [3, 6–10], especially for older adults, as it may exacerbate or may be exacerbated by their many comorbidities. In older patients, diabetes agents are implicated in 1 in 5 ED visits for ADEs [11] and at least one episode of hospitalization for hypoglycemia is associated with subsequent re-hospitalization and mortality [3]. For older adults with diabetes, a history of severe hypoglycemic episodes is also associated with a greater risk of dementia [12], fall-related fractures [13], and decreased quality of life [14]. Therefore, prevention of hypoglycemic events should be considered a high priority in older individuals.

In order to develop successful interventions to reduce hypoglycemic events, it is important to understand the underlying causes of severe hypoglycemia. Although multiple predisposing factors for hypoglycemia have been reported, such as lack of access to food [15–21], social vulnerability [17, 21], lack of support or knowledge [15], and treatment errors [3, 9], precipitating factors for hypoglycemia are often under-reported in the literature [15]. In a national study examining insulin-related hypoglycemia and errors leading to ED visits, precipitating factors for insulin-related hypoglycemia were documented in only 20.8% of cases [3], indicating that the majority of precipitating factors are still left to be identified. Furthermore, professional organizations and the US Department of Health and Human Services have called for data to better understand and thus prevent hypoglycemia [1, 22].

One step toward understanding the underlying causes for hospitalizations of hypoglycemia is to elicit the perspectives of patients on the reasons for their hypoglycemia. Accordingly, using qualitative methods, we conducted interviews with older patients with diabetes admitted to the hospital for severe hypoglycemia to investigate this complex phenomenon and to explore its cause(s).

## Methods

### Overall study design

We performed a qualitative analysis using in-depth interviews with older patients with diabetes admitted to the hospital with hypoglycemia. We chose qualitative methods because they are well-suited for investigating complex phenomena with multiple potential causes [23].

### Participant recruitment

Between October 2014 and February 2016, we recruited patients from Yale-New Haven Hospital (a large, tertiary referral teaching hospital located in an urban setting on the east coast) and its affiliate, Saint Raphael’s Hospital. We used electronic medical records to identify patients with diabetes, who were 65 years or older, and who were admitted with a primary or secondary diagnosis of diabetes-related hypoglycemia defined based on ICD-9 and ICD-10 codes. Following a careful review of the medical records to confirm the patient was actually admitted for hypoglycemia, potential participants were screened for delirium and dementia with validated tools (CAM, Confusion Assessment Method, and the Mini-Cog, respectively) to rule out significant cognitive impairment. Patients were considered eligible if they did not have delirium or significant cognitive impairment, had the capacity to participate in the study as assessed directly by the research staff, did not have severe hearing impairment, and were not admitted from a nursing home. All participants that passed the screening process and agreed to participate provided written informed consent. The study was approved by the institutional review board at Yale University.

### Data collection

Data were collected from in-person one-on-one in-depth open-ended interviews to explore participants’ experiences with diabetes management and hypoglycemia. Interviews were conducted during the admission so as to limit recall bias. Interviews were conducted using a standard qualitative interview guide, but allowing participants to direct the course of discussion as much as possible. The interviews began with a broad “grand tour” question [24], “Tell me about the reasons you were admitted to the hospital this time.” The interview guides explored the reason for admission, challenges experienced dealing with diabetes, medications and adherence, home life, financial issues, transportation, depression/cognitive function, functional disability, contact with the provider, and how the admission could have been potentially avoided. Supplementary Fig. 1 in Additional file 1 contains the interview guide that was used.

In addition to the established questions, the interviewer used prompts and probes to clarify concepts, elicit detail, and extend the narrative. All interviews were professionally recorded and transcribed. Additional data were collected from the review of the hospital electronic medical record, including comorbid conditions and medications used prior to admission.

### Data analysis

We analyzed the data using the constant comparative method [25], where coding and analysis took place simultaneously in order to systematically extract major themes that were then used to develop a theory [26]. Verbatim interview transcripts were coded using an iterative, inductive process by an interdisciplinary research team consisting of a medical student (W.P.), an organizational psychologist (D.B.), and an endocrinologist specializing in hypoglycemia (K.L). A codebook was developed to capture the emerging themes and was updated whenever new themes emerged or new connections between themes were discovered.

Each of the 3 coders individually coded the transcripts and then as a team, discussed data interpretation and thematic emergence, negotiated a consensus on the major themes, and identified potential sources of individual bias. Albeit rare, disagreements between coders were thoroughly discussed and clarified using exemplar quotes and concrete textual examples. Ultimately any disagreements led to a constructive re-evaluation of the codebook and major themes.

Several techniques were used to ensure the scientific rigor of our study: consistent use of an interview guide, professional audiotaping and transcription, the use of researchers with diverse professional backgrounds, an initial calibration period to ensure inter-rater reliability, and an audit trail to document all analytic decisions.

## Results

We conducted interviews with 17 patients, aged 65 years or older admitted to a single hospital with severe hypoglycemia. The mean age of study participants was 78.9 years of age (Table 1). Patients were predominantly female, African American, multi-morbid (with an average of 13 other chronic conditions aside from diabetes), and all but three used insulin to treat diabetes prior to admission. Only two patients were taking metformin and/or another oral diabetes medication, however, this was in addition to insulin.

**Table 1:** Characteristics of Study Participants

	*N* = 17
Age, mean (SD)	78.9 (9.3)
Sex	
% Female	76.5%
Race	
% White	35.3%
% African American	64.7%
Marital Status	
% Married/Partnered	17.6%
% Divorced/Separated	17.6%
% Widowed	52.9%
% Never Married	0.1%
% Unknown	0.1%
Number of Chronic Conditions, mean (SD)	13.3 (6.0)
% with Chronic Kidney Disease	41.2%
% with Heart Failure	52.9%
% with ≥5 Chronic Conditions	76.5%
Type of Diabetes Regimen	
% Insulin	64.7%
% Insulin + Sulfonylurea	17.6%
% Sulfonylurea	17.6%

### Patients reported

#### Surprise at severe hypoglycemia: “It just happened all of a sudden”

Initially, when patients were asked to tell the story of how they came to be admitted to the hospital, many explained that “it just happened all of a sudden.” Many also expressed that they did not have a complete understanding of how they ended up in the hospital, especially since they did not break from their usual routine that day and were doing everything “right.”

*“I wish I knew what happened. It was like a nightmare. I couldn’t believe it and then, of course, I’m saying, oh, my God! What happened to me here?”* (female, age 90s).

*“And I’ve had sugar problems for many years. But always controlled. And that’s why it was farthest from my mind … This was a big disappointment, a big surprise”* (male, age 90s).

Even though patients were largely aware of the symptoms they experience with hypoglycemia, patients were surprised this time around, for a variety of reasons. Some did not experience any warning signs of low blood sugar, others could not attribute their current symptoms as warning signs of hypoglycemia, and a few experienced symptoms too late (i.e. while they were already collapsing onto the floor).

*“It just happened all of a sudden. Usually I get like, I feel warm and I feel clammy. I didn’t feel a thing … “* (female, age 80s).

#### Adequate support, knowledge, and preparedness for hypoglycemia: “I always keep orange juice around the house”

One participant summarized our preconceptions about the characteristics of patients who experience severe hypoglycemic hospitalizations. She lamented:

*“I feel sorry for like elderly people who don’t know how to take their insulin or their medication for that matter. They can’t read, they can’t see it too well and there are many people like that and I don’t know who manages for them, a lot of them just struggle along themselves, by themselves until something happens to them”* (female, age 80s).

However, we found no evidence to support these preconceptions. Participants were knowledgeable about diabetes and hypoglycemia and elaborated on their support systems, such as visiting nurses, responsive physicians, and family or friends:

*“They have a nurse around the clock, and she’s really good with diabetes. You know, you ask her some questions...she really will give you the right answers”* (female, age 70s).

Even when probed, patients did not identify financial concerns, such as difficulties affording medications or food:

*“And with the medicine … everything is generic now. So that’s not as expensive as it used to be years ago”* (female, age 70s).

Upon probing, many patients expressed awareness and understanding of severe hypoglycemia along with preparedness for a hypoglycemic event, however, sometimes patients did not have a chance to employ their preparedness plan.

*“I ask them to bring me a bar candy cause my nurse says you need to keep a bar candy so if your sugar go down you can eat it. And I tell her, my sugar went down all right. And the candy is still there”* (female, age 70s).

#### Challenges balancing a diet to avoid hyperglycemia, while minimizing hypoglycemia: “I’m fighting two battles here”

Many patients initially attributed their hypoglycemic event to not eating enough, either in general or to match their insulin intake. This ranged from:

*“I think that I didn’t eat enough for the insulin I was taking”* (female, age 80s).

to

*“So evidently what I ate was not enough.” “What did you eat that day?” “I had 2 sausages. I had cheese and eggs and I had grits and I had a hot cup of tea”* (female, age 60s).

Patients cited various reasons for “not eating enough,” such as lack of appetite or not getting a chance to eat as the hypoglycemic event was already underway. No patients attributed “not eating enough” to an inability to access or prepare food.

Patients disclosed that one of the greatest challenges about managing diabetes is avoidance of foods that raise their blood sugar. One patient detailed her husband’s role in the delicate balance between weight maintenance and the avoidance of low blood sugar:

*“I didn’t want to eat too much because I didn’t want to gain weight because my husband gets really upset if I gain a pound or two. He’s not thinking about the other, so, I’m fighting two battles here”* (female, age 80s).

Even when probed about how to avoid future hospitalizations, patients discussed the need to resist tempting (high-glycemic index) foods and, ironically, expressed the need to focus more on the prevention of high blood sugar, rather than prevention of lows:

*“Keep my mind set on my insulin and keep my mind set on my meals. I’m probably going to have to watch what I eat cause temptation is strong …*” (female, age 70s)*.*

#### Belief that hyperglycemia necessitates medical intervention, but hypoglycemia does not: “When you feel dizzy and all out of it, 70- you drink your orange juice; 300, you call the doctor”

When prompted, some patients expressed a recent, disquieting history of lows.

*“For the last month or two, it’s been running kind of low.” “And what’s low?” “Low is 25, 30”* (female, age 60s).

Alarmingly, some patients recalled that they were previously hospitalized for severe hypoglycemia, but no changes were made to their regimen:

“*Did you end up in the hospital then?” “Yeah” “And did they change your insulin dose around then?” “No, no”* (female, age 70s).

Despite these oversights, patients predominantly described a responsive, supportive healthcare system. Many patients detailed their clinician’s attentiveness to their HbA1c along with a thorough understanding of the expectations for managing their high blood sugars:

*“I have a very good doc … and he’s very strict and he keeps me on the amount of insulins I should take”* (female, age 70s).

*“And my doctor told me … she said but never let it be over 200 in the mornin’”* (female, age 70s).

However, when asked if they share their blood sugars or discuss lows with the healthcare team, patients expressed that they do not need to and can handle low blood sugars on their own:

*“No! Because they take the A1c and they find it a little high but acceptable. I think it’s about seven. It should be six or lower. So it’s acceptable”* (male, age 90s).

*“What have they told you about low blood sugar reactions?” … “What I’m supposed to do. If it’s low drink some orange juice or get somethin’ sweet.” “What about to prevent them from happening?” “I don’t know”* (female, age 70s).

*“When you feel dizzy and all out of it, 70- you drink your orange juice; 300, you call the doctor”* (female, age 60s).

#### Tension between clinician-prescribed treatment plans and self-management: “I should have been doing it my way”

When probed more generally regarding their diabetes regimen, patients rarely reported lack of support from the healthcare system or difficulties in obtaining, accessing, or administering their medications. However, some patients felt that they should be doing something differently from the regimen they were prescribed:

*“I knew it was too much insulin to take … I should have been doing it my way”* (male, age 80s).

*“I didn’t realize I was taking 4 types of diabetes pills. No wonder I felt the way I did … it’s not safe taking these medications”* (female, age 60s).

“*… she told me to take 8 all the way across, 8 for breakfast, lunch, and dinner. So, I, that’s why I took the 8.” “I was on a sliding scale … before. Now, if my sugar, glucose was low, I wouldn’t take anything …”* (female, age 80s).

Only one patient described a treatment error; she was prescribed prednisone while in the hospital, but never got a chance to pick up the prescription because it was sent to the wrong pharmacy. She explained:

“*… this year I just started really being bothered with a lot of low sugars because I wasn’t on no prednisone and I was just taking my regular medication and my insulin”* (female, age 70s).

## Discussion

In this study of older adults with diabetes admitted for severe hypoglycemia, participants did not report the previously described reasons for hypoglycemia, including lack of access to food [15–21], or lack of supports for diabetes management from patients’ families [15] as well as our hypothesized reasons, such as lack of support from the healthcare system and patients’ lack of knowledge about how to treat hypoglycemia. Although treatment errors are thought to be major contributors to hypoglycemia among older persons [3, 9], only one participant described a medication mix-up.

Instead, we found evidence for a pervasive emphasis on the avoidance of hyperglycemia by both patients and the medical profession. In our study, older patients who had long-standing diabetes, good knowledge of hypoglycemia treatment, and several instances of recent hypoglycemia, still reported surprise at their hypoglycemic events. Many reported they were “doing everything right,” and we suggest that “doing everything right” is deeply entrenched with the prevention of hyperglycemia, rather than hypoglycemia. This was also evidenced by patients’ preoccupation with the avoidance of foods with high-glycemic indices that would cause hyperglycemia, even after they acknowledged that perhaps not eating enough food is what caused hypoglycemia. This emphasis on hyperglycemia was also echoed in patient experiences with the healthcare system; patients detailed their clinicians’ views on bringing down their HbA1c and a “top number” to avoid, while explaining that they rarely reported low blood sugars to their healthcare team and instead dealt with hypoglycemia themselves. This attention focused on glycemic control may have contributed to surprise at the occurrence of hypoglycemia, or neglect of preceding milder hypoglycemic events.

Although efforts are underway to shift from the prioritization of reaching pre-specified glycemic targets to a more balanced approach [27], our results suggest that the healthcare system’s previous emphasis on avoidance of hyperglycemia has been internalized by many older patients with diabetes. Participants in our study still prioritized the prevention of hyperglycemia over hypoglycemia. The emphasis on avoidance of hyperglycemia is further entrenched by existing quality measures targeting hyperglycemia, with none that specifically measure hypoglycemic events [28]. As a result, clinicians may be unaware of their patients’ hypoglycemic events [29], and our findings suggest that patients may be reluctant to report low blood sugars to their clinicians because they deem them as less likely to require medical intervention than hyperglycemic events. To correct this unbalanced approach and address this gap in communication, we propose that the healthcare system makes hypoglycemia a “medical” priority; improve communication strategies about the risks of hypoglycemia; include severe hypoglycemia as a quality measure for clinicians; and dedicate as much time, focus, and monitoring to low blood sugars as is typically dedicated to high blood sugars in older patients at risk for severe hypoglycemia.

Another major theme in our study was the tension in self-management of diabetes: between patients’ desire to adhere to prescribed treatment plans and their need to adjust treatment based on their experience and changes in their diet or activity. Some patients reported that they needed to eat just to compensate for the insulin they were taking. Many participants may not have felt empowered to share concerns about their treatment with the healthcare team or to make changes to their regimen when they felt something was amiss. This disconnect highlights a tension around where the control of self-management resides: the patient or the clinician. Perhaps with good intentions and supported by current recommendations to simplify complex regimens [6, 30, 31], physicians recommend treatment plans that are less flexible, but simpler to implement. However, the lack of flexibility and patient control may in fact predispose patients to severe hypoglycemia. In contrast, structured education programs studied in type 1 diabetes, like DAFNE, have allowed patients to retain more control and flexibility in their self-management [32, 33], while also improving glycemic control [32, 34, 35] and reducing severe hypoglycemia [4, 35, 36]. Due to a lack of existing literature [4], we propose that further research should expand upon the role of structured education programs on the management of type 2 diabetes and severe hypoglycemia, while also addressing the prevalence of self-management challenges in older adults who experience severe hypoglycemia.

It is also possible that clinicians make assumptions about patients’ capacity to self-manage their disease. In previous qualitative studies, healthcare professionals cited concerns regarding the suitability of self-management for patients they deemed not capable [37, 38]. With our predominantly female, African-American, urban population, we cannot discount the influence of gender, race, and socioeconomic status in clinicians’ assessments of patients’ capacity to self-manage. There is a paucity of literature focused explicitly on bias and self-management, but previous studies have reported differences in physicians’ perceptions of black vs. white patients, with black patients more likely to be seen as a risk for noncompliance [39] and experiencing shared-decision making less often than white patients [40]. Therefore, in order to develop safe and effective management plans, we propose that clinicians work closely with patients to create plans that “fit” – plans that match patients’ willingness and capacity for self-management, while tempering assumptions regarding patients’ capability to self-manage.

### Limitations

Our study has several limitations. Participants were recruited after hospital admission for severe hypoglycemia; therefore, our findings were unable to capture the hypoglycemic events that were managed in the outpatient setting or outside of the healthcare system. Our sample consisted of predominantly African-American, female participants from New Haven, so our results may not be representative of all older adults with diabetes who have experienced severe hypoglycemia. Furthermore, we did not collect data on all of the potential sociodemographic factors that could have influenced our findings. Although certain themes likely transcend differences in socioeconomic status, such as the idea that the prevention of hyperglycemia is more important than the prevention of hypoglycemia, the lack of food insecurity and medication mix-ups could potentially be explained by a patient population of higher socioeconomic status. However, our findings did achieve our aim of richly capturing the experiences of older patients with severe hypoglycemia admitted to Yale Hospital and its affiliate, St. Raphael’s. The last limitation is inherent to the methodology of qualitative work; the intent of our study, was not to prove or refute hypotheses but to generate them. We propose that further quantitative research is needed to assess the prevalence of the themes that emerged in our study and to evaluate the efficacy of potential interventions.

## Conclusions

Results from our study suggest that older patients with diabetes who have experienced severe hypoglycemia may hold an internalized over-emphasis on hyperglycemia. In addition, these patients may experience several challenges with self-management that were previously under-reported, such as maintaining a diet that avoids hyperglycemia and minimizes hypoglycemia; the idea that hypoglycemia is something that should be handled at home, while hyperglycemia necessitates medical intervention; and tension between clinician-prescribed treatment plans and patients’ ideas about self-management. Clinicians should work with patients to ameliorate some of these challenges in self-management and to shift from an ongoing emphasis on the prevention of high blood sugar, towards a more balanced, patient-centered approach in order to better treat older patients with diabetes at risk for severe hypoglycemia.

## Supplementary Information

**Additional file 1.** The additional file contains the “Sample Interview Guide” that was used to perform the interviews with patients enrolled in our study.

## Data Availability

The dataset analyzed in the current study is not publicly available as it consists of verbatim interview transcript PDFs, but is available from the corresponding author on reasonable request.

## Abbreviations

HbA1c: Hemoglobin A1c
ADE: Adverse drug event
ED: Emergency department
ICD: International classification of disease
CAM: Confusion Assessment Method
SD: Standard deviation
DAFNE: Dose Adjustment for Normal Eating
