# Supplementary material for: Qualitative analysis of reasons for hospitalization for severe hypoglycemia among older adults with diabetes
Source: BMC Geriatr. 2021 May 17;21:318. doi: 10.1186/s12877-021-02268-w (PMC8130109; doi:10.1186/s12877-021-02268-w)
Supplement: Supplementary file 1 — Additional file 1. The additional file contains the “Sample Interview Guide” that was used to perform the interviews with patients enrolled in our study. [file 12877_2021_2268_MOESM1_ESM.docx]

**Additional File 1 - Sample Interview Guide**

**BROAD QUESTIONS ABOUT REASONS FOR ADMISSION:**

1. Can you tell me the story of how you came to be admitted to the hospital?
2. Why do you think this happened to you?
3. Has this happened before? How was this time different?
4. What do you think could have been done differently?
5. What do you think you or others could do to help you avoid a hospitalization like this in the future?

**MORE SPECIFIC/PROBING QUESTIONS:**

1. What are the hardest things about managing your diabetes?
2. Can you tell me how obtaining and taking medications affects the management of your diabetes?
3. Do you check your blood sugars? Do you have any difficulties with checking your blood sugars? What are they?
4. Can you tell me about any financial difficulties you may have experienced managing your diabetes? Do you have difficulties obtaining enough food?
5. Can you tell me about any difficulties you may have had with transportation to and from medical appointments or to and from the pharmacy? Do you have trouble getting where you need to go?
6. Do you have any mood or memory problems? Can you tell me how they may affect how you manage your diabetes?
7. Do you have a nurse or a doctor who helps you manage your diabetes? Tell me about him/her. How is your relationship? Have you ever discussed low blood sugar reactions with him/her?
8. Tell me about your communication with your provider.
9. Are there other persons who help you with your diabetes? How do they help you?
10. What other information would you like to share with me?
